# Large thoracic defect due to shotgun violation – surgical emergency management

**DOI:** 10.3205/iprs000116

**Published:** 2017-08-21

**Authors:** Holger Rupprecht, Katharina Gaab

**Affiliations:** 1 Klinikum Fürth, Department of Visceral, Thoracic, and Vascular Surgery, Fürth, Germany

**Keywords:** shotgun injury, tension pneumothorax, damage control, deadly triad, emergency thoracotomy

## Abstract

Shotgun injuries from a short distance (<3 m) may cause massive bleeding and tissue destruction. Only immediate aggressive (surgical) therapy prevents lethal outcome.

We report about a 27-year-old patient, who was wounded on the left chest wall by a straight-cut shotgun from a short distance. In cases of this special traumatic pattern damage control measures are necessary. The measures should take place in preclinical emergency management (by the on-site emergency physician).

We report about the emergency management from admission to our hospital and the following surgical treatment until discharge from the hospital.

## Zusammenfassung

Nahschussverletzungen mit Schrot (Abstand <3 m) können zu massiven Blutungen und Gewebedestruktionen führen. Nur durch eine rasche und aggressive (operative) Therapie ist noch eine Rettung möglich.

Wir berichten über einen 27-jährigen Patienten, dem aus kurzer Distanz mit einer abgesägten Schrotflinte in die linke Brustwand geschossen wurde. In diesen speziellen traumatischen Fällen sind „damage-control“-Maßnahmen notwendig. Diese Maßnahmen müssen bereits vor Ort im Rahmen der Notfallversorgung (Notarzt) erfolgen.

Wir berichten über den seltenen Fall einer Nahschussverletzung mit Schrot und das Notfallmanagement vor Ort und die weitere Therapie im Krankenhaus.

## Introduction

Violations caused by potshots with shotgun pellets lead to massive tissue destruction (distance <3 m). Commonest the victims die from the caused bleeding. Only through a quick and aggressive (surgical) therapy rescue is possible [1], [2].

## Case presentation

A 27-year old patient was wounded on the left chest wall by a straight-cut shotgun from a short distance. After emergency intubation, placement of a compression bandage on the massive bleeding thoracic wound as well as placement of a thoracic drainage (28 Ch) in a peripheral hospital, the victim was transferred to our hospital by helicopter.

**Figure 1:**
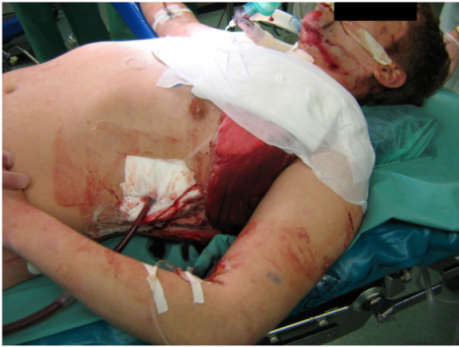
Tamponade and drainage of the left chest

**Figure 2:**
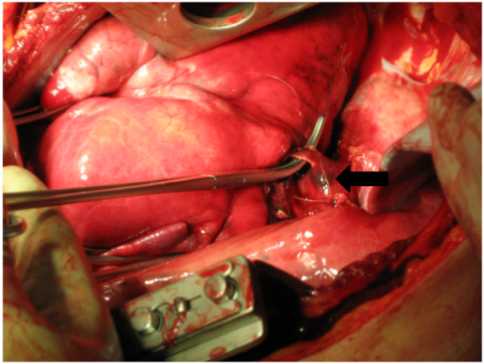
Pellet in the pulmonary artery (see arrow)

Upon arrival at the emergency trauma room a blood-soaked dressing showed. Through the drainage one liter of blood emerged and the bleeding was persisting (Figure 1 (Fig. 1)). Despite of pressure infusion of two red cell concentrates and several liters of ringer’s solution circulation could not be improved. That is why the patient was transferred to the operating room. During intubation with a double-lumen tube the blood pressure decreased and ventilation of the patient became difficult. On suspicion of a spontaneous tension pneumothorax on the left side (auscultatory breathing sounds only on the right side) a drainage was placed immediately in the second intercostal space on the left side (28 Ch). Air and blood emerged explosively through this drainage. After that, blood pressure increased and the ventilation improved (decrease in ventilation pressure). Simultaneously, sonography of thorax and abdomen was performed to exclude pericardial tamponade and intraabdominal bleeding. After left-lateral thoracotomy in the 5^th^ intercostal space there was a pleural cavity filled with clots of blood and a multiple perforated superior lobe with multiple contusions could be seen. From the shot hole blood poured massively. Additionally, a sagittal through-and-through wound in the inferior lobe and a shotgun pellet in the main stem of the pulmonary artery showed (Figure 2 (Fig. 2)). For bleeding control and prevention of pulmonary embolism the hilum, of the lung was immediately disconnected and suction was performed via cell saver. Because of the persistent diffuse bleeding, the pleural cavity was plugged temporarily with operating cloths. After quick resection of the superior lobe and release of the clamp on the hilum of the lung, the specific disconnection of the inferior lobe followed. The big shot channel was opened via linear cutter (“tractotomy”) and open vessels and bronchial tubes were ligated. Smaller superficial lesions could be sealed by argon beamer. The removal of the shotgun pellet from the pulmonary artery required a brief disconnection with following sewing-over with felt-laminated sutures. After removal of additional shotgun pellets from the hemorrhagic dorsal wall of the thoracic cavity, the prophylactic incision of the pericardium followed to exclude a lesion of the heart. 

At this time, the so-called “deadly triad” was already present with a massive hypothermia (31°C), a severe acidosis (pH=6.9) as well as a significant coagulation disorder (PTT=120 sec (normal parameters 26–36 sec.)). This fatal situation was only manageable through damage control measures, especially through “packing” of the pleural cavity with operating cloths. At this time 28 red cell concentrates and 12 units of fresh frozen plasma were given for stabilization of circulation. After loose adaption of the muscles with a continuous suture the bullet hole was debrided and several shotgun pellets were removed. Because of the coagulation disorder the wound was only plugged. After 24 hours of stabilization on the intensive care unit (correction of hypothermia and acid-base balance etc.) the tamponades could be removed and the thoracic cavity could be closed definitively with enclosed thoracic drainage. A necrotomy of the big chest wall defect followed and was afterwards supplied by a vacuum assisted closure dressing (VAC) with suction of 125 mmHg. After regular changing of the VAC dressing the wound could be covered with mesh graft transplantation after 3 weeks. After 4 months, the patient was released without any secondary failures. 

## Discussion

**Figure 3:**
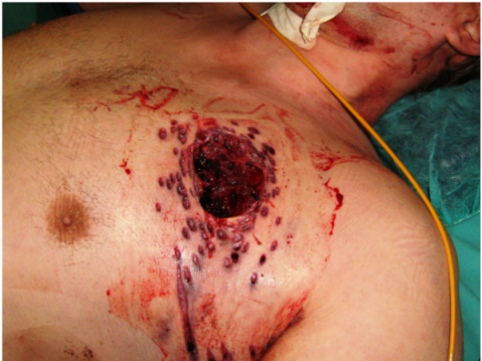
Large thoracic wound due to a shotgun

Shotgun injuries, especially from close distance (<3 meters), cause complex injury patterns with high lethality [3]. Next to partly massive tissue destruction with tearing of muscles (Figure 3 (Fig. 3)) and fractures of ribs, the massive bleeding, additionally accorded to trauma of parenchyma and vessels, leads to death in a short time [1], [2]. Ricochets generate particularly problematic impacts because they hit other body areas (e.g. contralateral thorax or abdomen) and cause additional damages [3], [4].

**Figure 4:**
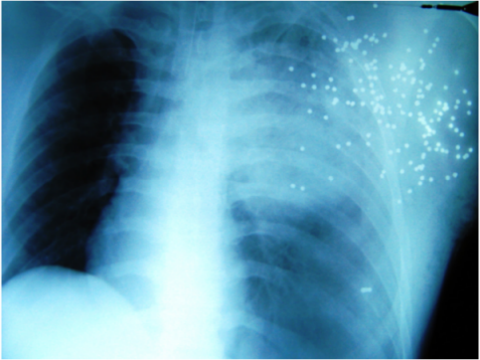
X-ray: multiple pellets in the chest

Special complications of shotgun injuries are “projectile embolizations”, which can lead through vessel obstruction to gangrene of lung, cerebral infarction or to vessel arrosion combined with a massive bleeding [5], [6], [7], [8]. To avoid these fatal consequences, a surgical resection is always indicated [5], [8]. Upon arrival at the emergency trauma room the X-ray of the thorax (Figure 4 (Fig. 4)) provides important information. Intraabdominal bleeding increases lethality dramatically [9]. To exclude a pericardial tamponade or intraabdominal bleeding (“free fluid in the abdominal cavity”) a sonography (FAST) of abdomen and thorax should be performed simultaneously [3], [10], [11], [12] [13]. The route of the ricochets determine the operative access [9], [12], [14]. For example in cases of heart or abdominal injuries, median sternotomy and median laparotomy are performed while in cases of a ipsilateral lung injury a lateral thoracotomy is performed [12], [14]. If the patient is haemodynamically stable, whole-body CT scan is the best diagnostic tool for detection of foreign bodies [4]. If there is not enough time for this investigation, because the patient is haemodynamically unstable, the whole-body CT scan has to be performed postoperatively. In cases of emergency thoracotomy [15], the FAST can be performed in the operating room during anesthetic induction. Additionally, the detection of metallic foreign bodies can be performed via C-arm X-ray to determine the operative access on time [6], [14]. After thoracotomy, the immediate bleeding control is decisive. The disconnection of the lung hilum is the fastest control opportunity (because due to the massive bleeding the situs is confusing) and the risk of air embolism is reduced by this maneuver [6], [12], [14], [16]. After suction of blood (most suitable is the cell saver for blood recovery [17]), the selective disconnection of hemorrhage sources follows, e.g. from the lung parenchyma or big vessels. The hilum clamp should be used only for a short time because acute right heart failure could occur. The dimension of resection of the lung has to be limited, because lethality increases proportionally to the loss of parenchyma. A pneumonectomy for example is related to a 80% higher mortality [2], [16], [18], [19], [20]. With a so-called “tractotomy” the shot channels could be opened and supplied with a minimal loss of parenchyma [3], [6], [9], [11], [16], [18]. Essentially, even in cases of no external damage, a pericardiotomy has to be performed to exclude a myocardial lesion [6], [12], [15]. This maneuver is also important if a direct bimanual cardiac massage is necessary [6]. Normally the patients with such massive traumata develop the so-called “deadly triad” [21], [22] with metabolic acidosis, hypothermia, and disseminated intravascular coagulopathy with consequential diffuse bleeding, which is only manageable through intrathoracal tamponade (“packing”) [14], [16], [21], [22], [23]. After these damage control measures the thoracic muscles are only adapted and the pleural cavity is left open [24], [25] to prevent thoracic compartment syndrome [26]. Afterwards, the patient is stabilized in the ICU with correction of the pathophysiological derangement (acidosis, hypothermia and coagulopathy). In a second-look operation (SLO) after 24 to 48 hours [12], [22] the tamponades can be removed and the thorax can be closed definitively. Because of the massive tissue destruction and the inclusions of contaminated metallic and powder fragments, the wounds have to be debrided carefully [2], [27], [28] combined with a high-sensitive antibiotic treatment [3], [27]. An extensive exploration of the wound is also necessary because inclusions of clothes or plastic fragments can not be detected via X-ray. The placement of a vacuum-assisted closure dressing has proven especially valuable [28], [29], [30], leading to quicker wound cleansing and wound granulation compared to traditional wound dressings [28]. That is why remaining defects can be covered more quickly with mesh graft transplantation [27], [28], [29], [30]. 

## Conclusions

Massive bleeding caused by shotgun injuries from a short distance needs an immediate aggressive (surgical) therapy to prevent lethal outcome. These measures should take place in preclinical emergency management (by the on-site emergency physician). Next to intubation and intravenous volume supply the tamponade of the bleeding wound will initially operate predominantly to prevent bleeding to death. Afterwards, a wide lumen thoracic drainage (at least 28 Ch) has to be placed via “minithoracotomy” to prevent a tension pneumothorax caused by the tamponade (insertion of trocars is contraindicated) [31], [32], [33]. Nevertheless a tension pneumothorax can develop for example because of malposition of the drainage or obstruction through blood clots [31], [32], [33], [34], [35], [36], [37]. An increase in blood pressure and a decrease in oxygenation as well as a volume-resistant shock [34] are warning signs concerning this matter and should effect the placement of a second wide lumen drainage [14], [16], [33], [34]. Possibly a contralateral pneumothorax (breathing sounds?) or a pericardial tamponade are present. In case of this traumatic pattern damage control measures [12], [16], [21], [23] and emergency thoracotomy are almost always necessary [9], [14], [20].

## Glossary of abbrevations

CT = computed tomography 

FAST = focused assessment with sonography in trauma 

ICU = intensive care unit

## Notes

### Competing interests

The authors declare that they have no competing interests.

### Compliance with ethical requirements

Prof. Dr. med. Holger Rupprecht and Dr. med. Katharina Gaab state that the article is original, has not been submitted for publication in other journals and has not yet been published, neither wholly nor in part. The authors state that they are responsible for the research that they have designed and carried out; that they have participated in drafting and revising the manuscript submitted, whose contents they approve. 

In the case of studies carried out on human beings, they confirm that the study was approved by the ethics committee and that the patients gave their informed consent. 

They also state that the research reported in the paper was undertaken in compliance with the Helsinki Declaration and the international principles governing research on animals.
